# The Human Genome Organisation (HUGO) and the 2020 COVID-19 pandemic

**DOI:** 10.1186/s40246-021-00310-x

**Published:** 2021-02-10

**Authors:** Benjamin Capps, Yann Joly, John Mulvihill, Won Bok Lee, HUGO Committee on Ethics, Law and Society, and the HUGO Council

**Affiliations:** 1 Department of Bioethics, Dalhousie University, 5849 University Avenue, CRC Building, Room C-312, PO Box 15000, Halifax, Nova Scotia B3H 4R2 Canada; 2 McGill University, Montreal, Canada; 3 University of Oklahoma Health Sciences Center, Oklahoma City, USA; 4 Ewha Womans University, Seoul, South Korea

**Keywords:** Human Genome Organisation, COVID-19, Genomics, Public health pathogen genomics

## Abstract

This letter is the Human Genome Organisation’s summary reaction to the 2020 COVID-19 pandemic. It identifies key areas for genomics research, and areas in which genomic scientists can contribute to a global response to the pandemic. The letter has been reviewed and endorsed by the HUGO Committee on Ethics, Law and Society (CELS) and the HUGO Council.

Letter to the Editor

It is over a year since the Program for Monitoring Emerging Diseases first detected the “urgent notice on the treatment of pneumonia of unknown cause” given by the Wuhan Municipal Health Committee. We now know the cause was Severe Acute Respiratory Syndrome Coronavirus 2 (SARS-CoV-2). It is difficult to take stock, with projections suggesting the pandemic is far from over and knowing that the impacts on health and wellbeing, health systems, and economies will last for years. Many countries have experienced multiple sequential waves of infection; but even if we can only anticipate each wave being the last, a vaccine strategy and plans for an ethical rollout are at least on the horizon. Evidence-based treatments for very sick people are available, as long as the health system they are in has access to the treatments and capacity to provide care; and in countries where there has been effective leadership—responsive to ‘following the science’, promoting effective public health measures, and investing in effective testing—hospitalisations have often been manageable. In some places, through public health, community spread has been stopped. Across the globe, the pandemic has severely impacted national economies and the security of communities and households, but the social and economic inequality it has caused or exacerbated has not been shared by all [1, 2].^1^

The first genetic sequence of the virus was transmitted to the WHO on 11th January 2020 and was immediately made available on the GISAID platform. That sequence was necessary for urgent diagnostics: the first step in combatting the virus’ spread in real time. Rigorous analyses of the viral genome, along with associated clinical data, has improved knowledge of the disease and enhanced our clinical responses to it. The first vaccine licenced by a national body (in December 2020) uses a novel mRNA approach to express viral proteins to induce an adaptive immunological response. Gene editing will have a role in better, faster testing [4]^2^ and designer therapies [5]; and eventually, individual responses to treatments could be enhanced by pharmacogenomics [6]. Genomics has a role in predicting individual prognoses of COVID-19, but it is critically important to integrate social determinants of health into this equation.

We have also seen the rise of polemical ‘bad science’. With supremacists conspiring to use every opportunity to destabilise society for their own advantage, science has been misused to stoke eugenic and racist divisions, and isolate communities [7]. Public health responses to the pandemic have unfortunately highlighted class and racial divides in respect to health and economic priorities, which have been acutely felt by marginalised communities [8]. Politicisation has undermined public health [9]; such politicisation could hinder international cooperation, as it has done in previous zoonotic outbreaks [10]. Despite early promise of a global concord, nationalism of resources is inevitable [11]. It is in these respects, that the HUGO Council and HUGO Committee on Ethics, Law and Society supports the American Society of Human Genetics' denouncement of the use of genetics to promote populism, supremacy, and ‘bad science’ [12, 13]. We share their view that all knowledge must be used to “advance science, improve health, and benefit people everywhere”.

In respect to genomics, we have three comments:

First, sharing sequences on open platforms has enabled a global pandemic strategy allowing for rapid dissemination of data, while also being an opportunity for enhanced scrutiny and detection of its flaws [14, 15]. There are ethical pitfalls in some Open Science models, especially those in which capitalist platforms (e.g. Amazon, Apple, Google, and Facebook, as well as data analysis start-ups such as Palantir and Clearview AI) provide essential public health activities based on neoliberal goals, rather than for the public good [16]. Researchers should be cautious with respect to who they collaborate with and consider the ethical as well as scientific implications of their decisions to publish preprints, and the timing and place of publication [17]. Too quick, or careless dissemination, allows errors to propagate before they are detected by traditional review processes; and in these cases, responsible researchers must be just as quick to correct them [18]. HUGO must help align resources to strengthen ‘precision public health pathogen genomics’ through its committees and annual conference; as such, it may frame the open commons for sharing and collaboration in a way that progresses science and supports our responses to genomic vulnerabilities [19]. We should also look forward to the time when the pandemic has passed, and we are left with vast amounts of samples and data: these should be made available under conditions of global ethics and a model that advances genomics research equitably.

Second, there continues to be limited effective integration of environmental factors in public health pathogen genomics. COVID-19 is a novel zoonotic disease, and, like other emergent infectious pathogens originating in wild animals, we poorly anticipate their pandemic potential. The detection of variants of the virus in farmed animals during the pandemic highlights a link between human and animal health [20]. Yet, despite initiatives to strengthen pathogen surveillance, disconnects remain between researchers, public health agencies, and policy-makers in environmental matters. These silos undermine progress in sustaining healthy ecoservices, particularly in circumstances where human activities are known to cause zoonotic spillover [21]. Genomic sciences must also be integrated in all aspects of pandemic response, including building an innovative infrastructure for future comparative studies [22], as well as contributing to understanding the impact of COVID-19 on non-human animals [23].

Third, the ‘right to science’ strengthens *bona fide* discovery; therefore, HUGO could provide leadership in responding to scientific activities that prospectively obstruct or reduce social opportunities or capture of public resources. The pandemic—or ‘syndemic’—has shown how multiple biological and social interactions ‘increase a person’s susceptibility to harm or worsen their health outcomes’ [24]. A key factor of our emergence from the pandemic will be how to create and sustain global equity, but we have already seen how public health measures can generate social disparities, and urgent access to cutting edge care will be a privilege many will not be afforded. Already, cracks in future effective and fair access to drugs and vaccines are showing because of political opportunism. HUGO reasserts that ‘genomic solidarity’ entitles everyone to access the benefits of research, so that citizens and scientists are joint owners in discovery and therefore must share its opportunities [25].

As we endure the present pandemic together, many face additional social and economic challenges, and it is this that compelled members of HUGO to write this open letter. HUGO recognises the human genome is the common heritage of humanity, and therefore this pandemic should also be an opportunity to use it to support a global and collective response, inclusive of all nations and communities.

## Data Availability

Not applicable.

## Footnotes

1 At this stage, it is difficult to quantify the present and eventual impact of the pandemic on nearly every aspect of society. These affects have broadly been described in terms of economic disruption [2] and public health, including stopping the virus spread, caring for patients with severe COVID-19, and treatment and vaccine development and access; but also must include long-term illness caused by COVID-19, the pandemic’s effect on access to non-COVID-19-related health care, and consequences for freedom and wellbeing across a spectrum of work and education [1], and social networks (connections in person and online, as well as socio-political turmoil magnified on social media [3]).

2 Citations reference cutting-edge work being done as the COVID-19 pandemic evolves. The references are either seminal, a starting point, or ‘of the moment’, and provide early data, reports, or reviews supporting the statements of this letter.
